# Constructing an In Vitro and In Vivo Flow Cytometry by Fast Line Scanning of Confocal Microscopy

**DOI:** 10.3390/s23063305

**Published:** 2023-03-21

**Authors:** Xiaohui Zhao, Leqi Ding, Jingsheng Yan, Jin Xu, Hao He

**Affiliations:** 1 School of Biomedical Engineering, Shanghai Jiao Tong University, Shanghai 200030, China; zhaoxiaohui@sjtu.edu.cn (X.Z.);; 2 School of Electronic Information and Electrical Engineering, Shanghai Jiao Tong University, Shanghai 200240, China; dlq991118@sjtu.edu.cn (L.D.);

**Keywords:** biosensing, confocal microscope, flow cytometry, in vivo biosensor

## Abstract

Composed of a fluidic and an optical system, flow cytometry has been widely used for biosensing. The fluidic flow enables its automatic high-throughput sample loading and sorting while the optical system works for molecular detection by fluorescence for micron-level cells and particles. This technology is quite powerful and highly developed; however, it requires a sample in the form of a suspension and thus only works in vitro. In this study, we report a simple scheme to construct a flow cytometry based on a confocal microscope without any modifications. We demonstrate that line scanning of microscopy can effectively excite fluorescence of flowing microbeads or cells in a capillary tube in vitro and in blood vessels of live mice in vivo. This method can resolve microbeads at several microns and the results are comparable to a classic flow cytometer. The absolute diameter of flowing samples can be indicated directly. The sampling limitations and variations of this method is carefully analyzed. This scheme can be easily accomplished by any commercial confocal microscope systems, expands the function of them, and is of promising potential for simultaneous confocal microscopy and in vivo detection of cells in blood vessels of live animals by a single system.

## 1. Introduction

Flow cytometry is a widely-used technology in biosensing, cell sorting, and high-throughput molecular detection based on fluidic and optical systems. The key machinery of it is fluorescence detection of cells or microspheres in a stable flow [1,2,3]. In this way, by labeling cells with fluorescence, whose specificity is determined by the form of fluorophore combination, flow cytometry enables detection of intracellular molecules [1,4]. To this purpose, the cytometry system usually sets a collimated laser beam perpendicular to the fluidic flow to excite fluorescence of passing cells. To resolve the micron-level samples, the laser beam is shaped as a long light sheet, whose thickness is around 10 μm. The passing samples scatter the incident laser and emit excited fluorescence in the light sheet. Although the information of sample morphology is theoretically embedded in the fluorescence signal, i.e., the width of the fluorescence pulse given the fluidic speed is a constant, in practice, the morphology is extracted from the scattering light, even indirectly, to eliminate the error of fluctuation of the fluidic speed and the fluorescence labeling. Only the amplitude of fluorescence signals is counted as the indication of molecules [5].

The requirement of a cytometry system on such in vitro fluidic flow of cells prevents it from in vivo applications. Interestingly, fluorescence microscope systems are able to perform in vivo two-dimensional microscopy imaging of animals, but do not work for high-speed flowing cells. By setting up a laser sheet in vivo, the concept of in vivo flow cytometry (IVFC) has been proposed for flowing cell detection in live animals [6,7,8]. The blood flow was considered as a fluidic system while the cells needed to be fluorescently-labeled. This setup had been used for monitoring of circulating tumor cells in transgenetic animal models whose tumor cells were labeled by fluorescent proteins [9,10,11,12]. To improve the information, the idea of imaging flow cytometry (IFC) was also developed. By specially-designed fast imaging strategies, IFC systems captured bright-field and even fluorescent images of high-speed flowing cells in vitro [13,14,15,16,17]. A straightforward scheme was laser scanning cytometry (LSC) utilizing the microscope platform [18,19]. The cells adherent on glass slides moving across the laser scanning line were imaged by confocal microscopy rather than cell flow. Recently, IFC has been making great progress. The imaging speed has achieved more than 10^4^–10^6^ 2D images/s, by a series of distinct technologies, including temporally coded excitation imaging [20], spatial-temporal transformation imaging [21], optical time-stretch imaging [22], stimulated Raman scattering imaging [23,24], and recent intelligent imaging [25,26]. However, such custom-designed microscopic systems are costly and only work in vitro.

In this study, we report a quite simple flow cytometer that can be constructed by any commercial or homemade confocal microscope systems without any modification. This microscope-based cytometry system works for both in vitro and in vivo samples with high spatial resolution. Our results indicate the confocal microscope systems are naturally of good potential for more biosensing functions.

## 2. Materials and Methods

### 2.1. Materials

Microbeads used in this experiments (PSGF005000, PSGF01000, PSGF03000, PSGF03000, PSGF10000) were obtained from Zhongkeleiming (Beijing) technology Co., Ltd.

Human prostate cancer cell line PC3 cells with stable expression of green fluorescent protein (GFP) were cultured in Roswell Park Memorial Institute 1640 medium containing 10% fetal bovine serum, 2 mM L-glutamine, and 1% (*v*/*v*) penicillin/streptomycin at 37 °C with 5% CO_2_. 

Male BALB/c nude mice, age 8 weeks, weighing approximately 20 g were purchased from Charles River and maintained in a specific pathogen-free (SPF) environment. Animal care and experimental protocols were approved by the Institutional Animal Care and Use Committee (IACUC) and the Ethical Committee of Animal Experiments of School of Biomedical Engineering at Shanghai Jiao Tong University (Approval number: 2019036).

### 2.2. Line Scanning and Fluidic System

#### 2.2.1. Line Scanning

For in vivo experiments, the blood vessels of mice were injected intravenously with the dye Allophycocyanin (APC, 0.1 mg/mL, 100 μL), which emitted red fluorescence when excited by a 532 nm laser. The microspheres and PC3-GFP cells emitted green fluorescence when excited by a 488 nm laser. For the in vitro experiments, the microspheres at sizes of 500 nm, 1, 3, 5 and 10µm were dissolved in phosphate buffered saline at a concentration of 10^6^/mL, and pumped through the glass tube with a peristaltic pump at the speed of 2, 4, 6, and 10 µL/min. Under a 20× objective, we defined a transverse scanning line across the glass tube or an auricular vein vessel in vivo, which was mapped to 256 pixels with a dwell time of 2 μs for a continuous time-lapse loop. For each experiment, about 300 fluorescence images of 256 × 256 pixels were obtained by scanning the defined line over 10 min. The imaging plane of the confocal microscopy was tuned according to the actual position of the fluidic flow.

#### 2.2.2. Fluidic System

The fluidic system was set by a peristaltic pump that pumped the suspension of microbeads at 2–10 µL/min. The pump was connected to a glass capillary tube (100 µm in diameter) by a plastic pipe. The capillary tube was put on a glass slide on the microscope stage. Before experiments, pure water was pumped into this system for 15 min to evacuate the air inside to prevent bubbles. Then, the flow of microbeads was pumped into the tube and we performed microscopy to precisely tune the imaging plane on the flow. 

## 3. Results

**Figure 1:**
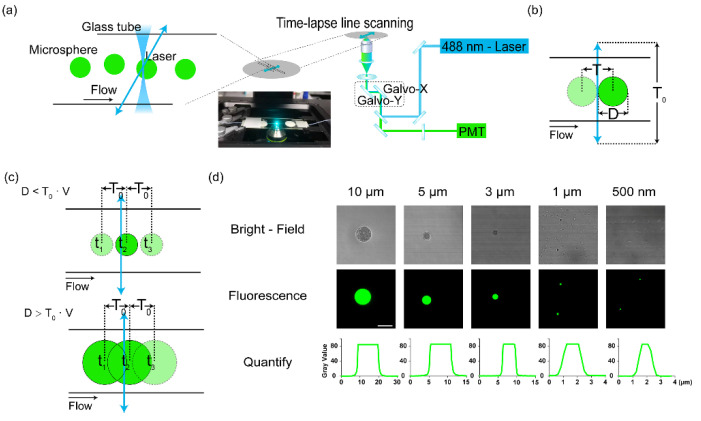
The scheme of line scanning by a confocal microscope for cytometry. (**a**) The setup of the line scanning method to detect flowing samples. The scanning line was defined transversely across the flow. The fluidic system used a peristaltic pump to pump suspensions of microbeads to a capillary tube (100 μm in diameter) that was put on a glass slide on the microscope stage. Arrow line: the laser scanning. (**b**) The scanning line transversely across the fluidic flow. D: diameter of the passing particles. T_0_: time of a single-time scanning period of the laser. (**c**) The sampling frequency was determined by the fluidic velocity (v). Upper panel: the case of less sampling: D < v∙T_0_; lower panel: D > v∙T_0_. (**d**) The quantification of diameter of different microbeads by their fluorescence by confocal microscopy. Bar: 10 μm.

To promote the confocal microscope working as a flow cytometer, we designed a scheme as shown in Figure 1a. The galvo-mirrors in the confocal microscope could control the excitation laser beam to scan in a region of interest, which was defined as a single line located in any target spatial position. The fluidic flow was accomplished by a capillary tube of glass. The suspension of cells or microbeads was directly pumped into it. The scanning line was defined transversely across the tube. The excitation lasers were then focused on it and repeated fast line scanning. In this way, the passing samples could be excited by the scanning laser beam, and the fluorescence was collected by the objective (Figure 1b). However, in this scheme, the transverse laser beam scanning was not a continuous stable light sheet. The moving laser focus along the line might have missed the fast-passing cells, or the sampling on a single cell was not enough (Figure 1c). Therefore, this scheme required the match of the flow speed (diameter of the sample) and the scanning speed to ensure enough sampling. There was a balance between the line scanning speed/frequency and the limit of the flowing speed of the sample (Figure 1c).

To establish the parameters of the scheme, we tested this method using a series of fluorescent microbeads (GFP green) at different diameters, from 0.5 to 10 μm (Figure 1d). The size of those beads was confirmed by confocal microscopy, excited by a 473 nm laser. The 500 nm beads were close to the measurement limit of microscopy. In the following study, we used 500 nm beads as the bottom line of the system.

**Figure 2:**
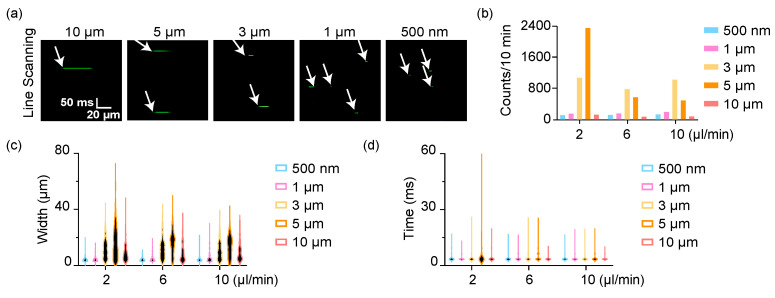
Detection of flowing microbeads by line scanning. (**a**) The time-lapse imaging of flowing microbeads. The images are stacks of the fluorescence of each line scanning. (**b**) The detection counts of each microbeads. (**c**) The width of microbeads directly indicated by the fluorescence in (**a**). (**d**) The scanning time of each microbeads.

We set the flow speed of the microbeads suspension from 2 μL/min to 10 μL/min (the flow line speed was from 4 mm/s to 20 mm/s accordingly. As a reference, the blood flow speed of each mouse was around 1 mm/s). The diameter of capillary tube was 100 µm. We defined the scanning line transversely across the glass tube, which was sampled by 256 pixels. The dwell time of each pixel was 7.8 µs, generally the same as for usual fluorescent confocal microscopy, which was enough for integration of fluorescence for most fluorophores. Here, it took 3.04 ms for a single-time line scanning circle, which could be tuned according to practical cases. The sampling speed of this scheme was then 329/s. We used an objective (20×, N.A. = 0.6) to get a submicron spatial resolution (~0.85 μm) and then performed a time-lapse microscopy for a 10 min continuous detection (or repeated the scanning for any defined times). The original fluorescent signals of those microbeads are shown in Figure 2a. The horizontal axis represents the spatial information (the scanning line) while the vertical axis is time. If the microbeads were smaller than 1 μm, the fluorescence intensity was quite low, and hard to capture, which further influenced the detection efficiency. The detected counts of those microbeads from our tests are shown in Figure 2b. The detected microbeads were approximately the same at different speeds except the 5 μm beads at 2 μL/min, which presented an ultra-high effective detection efficiency. However, the detection of other beads at different flow speeds variated slightly. The technology could directly indicate the absolute width of flowing beads but in a two-time manner, i.e., the detected width of the fluorescence signal included both the back-and-forth scanning, as well as the dead time at the return point of the galvo mirror (Figure 2c). Among them, the system could resolve beads from 1 μm to 5 μm, but could not distinguish the small beads at 500 nm from 1 μm. The width of 10 μm beads could hardly be detected properly due to the heavy beads flowed at the bottom of capillary tube. They were a little out of focus since the laser scanning mainly excited the chord in some cross sections in the upper half beads. The fluctuation of the flow also introduced some errors in this scheme (Figure 2d). In general, those data showed this scheme could detect the flowing beads in a fluidic flow system at several microns. The detection efficiency and accuracy could further be improved if setting a more stable fluidic system. 

**Figure 3:**
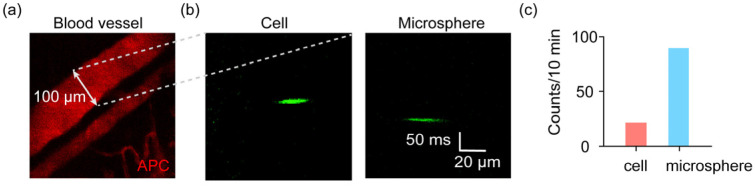
In vivo detection of injected microbeads and cells in live mice. (**a**) The fluorescence image of blood vessels indicated by APC. (**b**) The in vivo images of flowing cells and microbeads in a vein in the ear of a mouse. (**c**) The counts of detected cells and microbeads in a mouse.

We finally demonstrate the significant advantage that this system could work for in vivo samples. We injected the microbeads at 10 μm (GFP-labeled) and PC3 cells (GFP labeled, green) into live mice by tail vein injection. The mice were anesthetized and put on the stage of the microscope. We performed microscopy of blood vessels in the ear of each mouse, which was labeled by APC (red). A vein was randomly selected in one ear (Figure 3a). Accordingly, a scanning line was defined transversely across the vein to let the excitation laser scan the section of the blood vessel. The injected microbeads and cells circulated along the circulation system of the mice, within the natural fluidic system. In this way, the passing cells and microbeads could then be detected (Figure 3b,c). 

**Figure 4:**
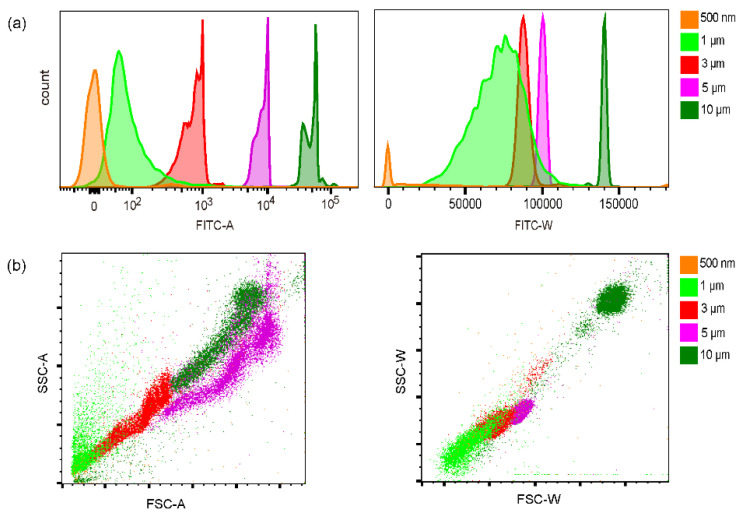
Quantification of microbeads by FACS Aria II flow cytometer. (**a**) The fluorescence amplitude and width. (**b**) The front scattering and side scattering signals.

We measured the fluorescence signals using a commercial flow cytometer (FACS Aria II, BD) as a comparison. In Figure 4a, the amplitude and width of fluorescence signals of each microbeads could be found. Interestingly, this flow cytometer could more or less resolve those beads by the fluorescence amplitude, although the absolute amplitude of the 0.5 μm beads was close to zero. The variation of 1 μm and 3 μm beads was also relatively large. The width of the fluorescence signals of 3, 5, and 10 μm beads presented little variation. However, the fluctuation of the fluorescence width of 0.5 and 1 μm beads was significantly larger than that of amplitude. By width, the 1 μm and 3 μm beads could not be resolved. The scattering signals of those beads variated a lot (Figure 4b). It was possible to distinguish the 3, 5, and 10 μm beads, but the scattering of 0.5 μm beads could not be detected. The variation of 1 μm beads was too large to extract them. Those results suggested the detection of the microbeads by our line scanning scheme was generally comparable to the result by commercial cytometry except the beads less than 1 μm. In this measurement, the absolute size of the beads could not be indicated.

## 4. Discussion

In this study, we present a simple scheme to construct an in vitro and in vivo cytometry by continuous temporal line scanning of confocal microscope systems without any modification. The laser was focused to a submicron diffraction limit in this method. Hence this scheme can theoretically resolve particles larger than 1 μm and provide direct measurement, not only distinguish them. The fast line scanning of excitation lasers in confocal microscopes can work as a dynamic light sheet to excite passing samples. According to the laser scanning speed along a 100 μm line that is around at the millisecond level for a single-time line scan period, to take enough sampling, there is a limit of the flowing speed, given that the sample is at the level of several microns. Otherwise, the fast fast-flowing beads or cells cannot get enough scanning times for valid detection.

Another limitation of this scheme is the excitation laser is focused by the objective (which also works for the fluorescence collection). The laser beam is thus not collimated at the penetration direction. Therefore, only flowing microbeads or cells exactly inside the imaging plane of microscopy can be effectively excited. The depth of the plane is determined by the focal length of the objective used. In this regard, this scheme is quite sensitive to the spatial fluctuation of the flowing samples at the vertical direction. This is the reason why the fluorescence signal of the 10 μm microbeads was not reasonable (Figure 2c), which generally flowed in a different plane from the small-sized beads or even fluttered in the capillary tube. The variation of the fluorescence signals from different microbeads also mostly comes from this, resulting in the variation of measured size of those beads. Therefore, in this demonstration, the fluidic system composed of a peristaltic pump and capillary tube greatly diminishes the performance of this scheme. A much better fluorescence detection can be expected if this method is promoted with a stable fluidic flow.

The scheme presented in this study can work both in vitro and in vivo. It can detect fluorescently-labeled cells in blood vessels of live mice and enable confocal microscopy and in vivo flow cytometry in the same system and even in the same field of view. This is thus a very convenient method to take advantage of confocal microscope systems to detect cells in circulating systems of animals, including, for example, circulating tumor cells (CTCs). The primary tumor transplanted to mice could be genetically labeled with fluorescent proteins. In this case, during tumor development, the CTCs could be monitored continuously in vivo, and the invasion and migration of CTCs from blood to organs could also be monitored under the same system that only requires changing to microscopy. The autofluorescence from skin is mainly from keratin molecules in the spinous layer in the epidermis. In this scheme, the excitation in blood vessels in dermis can thus avoid it. 

In general, this scheme is generally quite simple; it can be accomplished by confocal microscope systems directly without any modification, and provide accurate and powerful biosensing. We therefore believe it is of promising potential in in vitro and in vivo sensing as and beyond a flow cytometer.

## Data Availability

The data that support the findings of this study are available on request from the corresponding author.
